# Chitinase Genes *LbCHI31* and *LbCHI32* from *Limonium bicolor* Were Successfully Expressed in *Escherichia coli* and Exhibit Recombinant Chitinase Activities

**DOI:** 10.1155/2013/648382

**Published:** 2013-12-07

**Authors:** Zhihua Liu, Ying Huang, Rongshu Zhang, Guiping Diao, Haijuan Fan, Zhiying Wang

**Affiliations:** ^1^School of Forestry, Northeast Forestry University, 26 Hexing Road, Harbin 150040, China; ^2^The College of Landscape, Northeast Forestry University, 26 Hexing Road, Harbin 150040, China

## Abstract

The two chitinase genes, *LbCHI31* and *LbCHI32* from *Limonium bicolor*, were, respectively, expressed in *Escherichia coli* BL21 strain. The intracellular recombinant chitinases, inrCHI31 and inrCHI32, and the extracellular exrCHI31 and exrCHI32 could be produced into *E. coli*. The exrCHI31 and exrCHI32 can be secreted into extracellular medium. The optimal reaction condition for inrCHI31 was 5 mmol/L of Mn^2+^ at 40°C and pH 5.0 with an activity of 0.772 U using *Alternaria alternata* cell wall as substrate. The optimal condition of inrCHI32 was 5 mmol/L of Ba^2+^ at 45°C and pH 5.0 with an activity of 0.792 U using *Valsa sordida* cell wall as substrate. The optimal reaction condition of exrCHI31 was 5 mmol/L of Zn^2+^ at 40°C and pH 5.0, and the activity was 0.921 U using the *A. alternata* cell wall as substrate. Simultaneously, the optimal condition of exrCHI32 was 5 mmol/L of K^+^ at 45°C and pH 5.0, with *V. sordida* cell wall as the substrate, and the activity was 0.897 U. Furthermore, the activities of extracellular recombinant enzymes on fungal cell walls and compounds were generally higher than those of the intracellular recombinant enzymes. Recombinant exrCHI31 and exrCHI32 have better hydrolytic ability on cell walls of different fungi than synthetic chitins and obviously showed activity against *A. alternata*.

## 1. Introduction

Fungal phytopathogens are one of the major constraints in global food production as they cause many of the world's most notorious plant diseases [1]. Chitin is the main component of the cell walls of fungal plant phytopathogens and can be decomposed by chitinase. Plant chitinases expressed during plant and phytopathogen interactions are involved in defense responses of the host plant against pathogens [2].

The role of plant chitinases in protecting plants against a variety of fungal pathogens is well characterized. For instance, the overexpression of a chitinase gene (*McCHI1*) from *Momordica charantia * dramatically increased intercellular and intracellular endochitinase activities and significantly enhanced resistance to the plant pathogenic fungus, *Phytophthora nicotianae*, in transgenic *N. benthamiana* plants and against *Verticillium* wilt in transgenic cottons [3]. Overexpression of the chitinase gene, *BbCHI1*, from *Beauveria bassiana* enhanced disease resistance to *C. chrysosperma* in transformed poplar plants, which indicated that this gene is potentially useful in protecting these trees against fungal diseases [4]. The plants that expressed the endochitinase *CHI42* gene from *Metarhizium anisopliae* were consistently resistant to the soil-borne pathogen, *Rhizoctonia solani*, which suggests a direct relationship between enzyme activity and a reduction in the foliar area affected by fungal lesions [5]. The rice chitinase gene, *RicCHI11*, was transferred into Taro (*Colocasia esculenta*), and the resulting transgenic lines exhibited improved tolerance to the fungal pathogen *Sclerotium rolfsii* [6]. A chitinase gene *CHI30* from *Streptomyces olivaceoviridis* ATCC 11238 was transformed into pea and the transgenic pea inhibited the development of *T. harzianum in vitro* [7]. Transgenic wheat that expressed a barley class II chitinase exhibited enhanced resistance against *Fusarium graminearum* (*Fusarium* head blight) [8]. These studies suggest that plant chitinases are involved in plant resistance to pathogens; therefore, chitinase genes have potential uses in plant engineering programs to protect against fungal diseases.

To study the properties of recombinant plant chitinases, chitinase genes have been expressed in *E. coli* and yeast. The barley chitinase gene was expressed in *E. coli* and the purified chitinase exerted broad-spectrum antifungal activities against different phytopathogens, including *Botrytis cinerea* (blight of tobacco), *Pestalotia theae* (leaf spot of tea), *Bipolaris oryzae* (brown spot of rice), *Alternaria* sp. (grain discoloration of rice), *Curvularia lunata* (leaf spot of clover), and *Rhizoctonia solani* (sheath blight of rice) [9]. An acidic class VII chitinase gene from wheat has also been expressed in *E. coli* BL21. Purified chitinase exerted a wide antifungal activity against *Colletotrichum falcatum* (red rot of sugarcane), *Pestalotia theae* (leaf spot of tea), *Rhizoctonia solani* (sheath blight of rice), *Sarocladium oryzae* (sheath rot of rice), *Alternaria* sp. (grain discoloration of rice), and *Fusarium* sp. (scab of rye) [10]. A class IV chitinase gene *CpCHI* from papaya expressed in *E. coli* can completely inhibit spore germination in *Alternaria brassicicola* and also showed antibacterial activity [11]. *Pichia*-expressed *BjCHI1* from *Brassica juncea* showed antifungal activities against phytopathogens, *Colletotrichum truncatum, C. acutatum, Botrytis cinerea,* and *Ascochyta rabiei,* and also inhibited spore germination of *C. truncatum* [12]. These studies showed that plant chitinase genes could be expressed in *E. coli* or yeast and the resulting recombinant chitinases display high antifungal activities. Therefore, recombinant plant chitinases produced by *E. coli* or yeast have potential antifungal uses.

In the present study, *LbCHI31* and *LbCHI32* were, respectively, cloned into a prokaryotic expression vector pET52b and transformed into *E. coli* BL21 strain. Four different recombinant chitinases, intracellular inrCHI31 and inrCHI32 and extracellular exrCHI31 and exrCHI32, were produced with transgenic *E. coli*. The properties of these four recombinant chitinases were studied. Furthermore, the properties of LbCHI31 and LbCHI32 were analyzed. Our studies suggested that *LbCHI31* and *LbCHI32* have high levels of activity against fungal cell walls and may have antifungal applications in plants.

## 2. Materials and Methods

### 2.1. Strains and Plasmids

*Escherichia coli* strain Top10 (TaKaRa Biotechnology Co., Ltd., Dalian, China) was used for the genetic manipulation; *E. coli* BL21 strain and pET-52b(+) vector (Novagen, Madison, USA) were employed for the prokaryotic expression experiments. The six kinds of fungal/oomycete plant phytopathogens were *Rhizoctonia solani* (rice sheath blight), *Fusarium oxysporum* (soybean root rot), *Sclerotinia sclerotiorum* (sclerotium disease on soybean), *Alternaria alternata* (poplar leaf wither), *Valsa sordida* (poplar bark rot), and *Phytophthora sojae* (soybean root rot).

The protein families of the *LbCHI31* and *LbCHI32* genes were analyzed using the Pfam program (http://pfam.sanger.ac.uk/). The three-dimensional structures of the LbCHI31 and LbCHI32 proteins were identified by Geno3d (http://geno3d-pbil.ibcp.fr/cgi-bin/geno3d_automat.pl?page=/GENO3D/geno3d_home.html).

### 2.2. Primer Design

The two chitinase genes, *LbCHI31* and *LbCHI32* (GenBank numbers: DQ431248 and DQ431249), from *L. bicolor* were cloned into pET52b vector and transferred into *E. coli* BL21 strain. The primers for the construction of prokaryotic intracellular and extracellular expression vectors were designed and are shown in Table 1. For extracellular expression, signal peptide sequence of *LbCHI31* and *LbCHI32* has also been cloned into pET52b vector, respectively.

### 2.3. Vector Construction and *E. coli* Transformation

The ORFs of the two chitinase genes, *LbCHI31* and *LbCHI32*, were amplified using the corresponding primers and digested with double enzymes (Table 1), ligated into the expression vector of pET52b, and transferred into *E. coli* Top10F′ competent cells using the heat shock method. This resulted in four kinds of recombinant vectors that were designed as pET-inCHI31 (harboring intracellular expressed *LbCHI31*), pET-exCHI31 (harboring extracellular expressed *LbCHI31*), pET-inCHI32 (harboring intracellular expressed *LbCHI32*), and pET-exCHI32 (harboring extracellular expressed *LbCHI32*). The recombinant vectors were, respectively, transferred into *E. coli* BL21 strain to induce their respective expression.

### 2.4. SDS-PAGE Analyses

Four kinds of *E. coli* transformants (named BL21-inCHI31, BL21-exCHI31, BL21-inCHI32, and BL21-exCHI32) and control transformant BL21-pET52b (*E. coli* BL21 transformed with empty plasmid pET52b) were induced following the procedures in the Novagen manual (Cat. No.: 71554-3), respectively. IPTG was added into the LB medium at a final concentration of 1.0 mM to induce exogenous gene expression. The supernatants of the transformants BL21-exCHI31 and BL21-exCHI32 and the cells of the transformants BL21-inCHI31 and BL21-inCHI32 were harvested after they were cultured for 1, 2, 3, 4, 5, and 6 h at 30°C. After the addition of 1 × loading buffer, the supernatants or cells were boiled for 5 min, centrifuged for 10 min at 8,000 rpm, and loaded into a 12% slab gel.

### 2.5. The Detection of Recombinant Chitinase

To measure chitinase activity, the *E. coli* transformants BL21-inCHI31, BL21-inCHI32, BL21-exCHI31, and BL21-exCHI32 were induced by 1.0 mM IPTG at 30°C for 2 to 8 h, at 1 h intervals. The culture solution was centrifuged and the supernatant (enzyme solution) was used to measure the chitinase activity. The supernatant used as a control was boiled for 20 min at 100°C. To study whether the *E. coli* strain transformed with the empty pET52b also displayed chitinase activity, the strain transformed with empty pET52b (BL21) was induced by 1% (v/v) methanol at 30°C for 1 to 8 h at 1 h intervals as controls. Chitinase activities were measured according to the Schales procedure [13] with some modifications. In brief, the reaction mixture, consisting of 1 mL of colloidal chitin (1%, w/v) as the substrate and 1 mL of enzyme solution, was incubated at 37°C for 20 min, boiled for 5 min with the addition of 2 mL of 0.05% (w/v) KFe (CN), and then boiled again for 10 min. After cooling, the reducing sugars that were released as a response to chitinase activity were measured at 420 nm. One unit of chitinase activity was defined as the amount of enzyme that produced 1 *μ*g of reducing *N*-acetyl-D-glucosamine per minute.

The optimal temperature and pH for chitinase activity, thermal stability, and the effects of ions on enzyme activity, and the rate of decomposition of fungal cell walls of recombinant chitinases inrCHI31, exrCHI31, inrCHI32, and exrCHI32 were investigated according to the technique described by Liu et al. [14, 15].

All of the above experiments were performed in triplicate, at a minimum, and the average values were calculated based on the results of three independent experiments.

### 2.6. Antifungal Activity Analyses of the Two Recombinant exrCHI31 and exrCHI32

Recombinant exrCHI31 and exrCHI32 were mixed to PDA medium with the final concentration of 20 *μ*g/mL compared to the original concentration. Further, five millimeter mass of *A. alternata* was inoculated at the center of medium. And then, we are noted to inhibit affection by taking picture.

## 3. Results

### 3.1. Comparison of the Structures of LbCHI31 and LbCHI32 Chitinases

**Figure 1:**
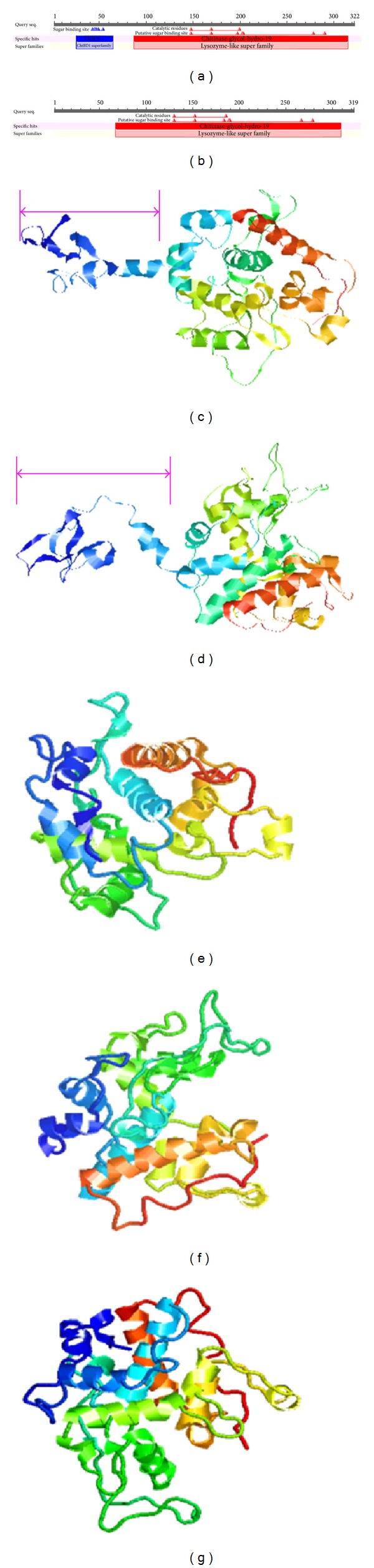
Protein families and the three-dimensional structures of LbCHI31 and LbCHI32 chitinases from *L. bicolor*. (a) Protein family of the LbCHI31 chitinase; (b) protein family of the LbCHI32 chitinase; (c-d) three-dimensional structure of LbCHI31 chitinase; (e–g) three-dimensional structure of the LbCHI32 chitinase.

The LbCHI31 and LbCHI32 chitinases both belong to the chitinase-glyco-hydro-19 family, and they both contain three chitin catalytic residues and six putative sugar binding sites (Figures 1(a) and 1(b)). However, only LbCHI31 was found to contain a chitin binding region (Figures 1(c) and 1(d)).

### 3.2. SDS-PAGE Analysis

**Figure 2:**
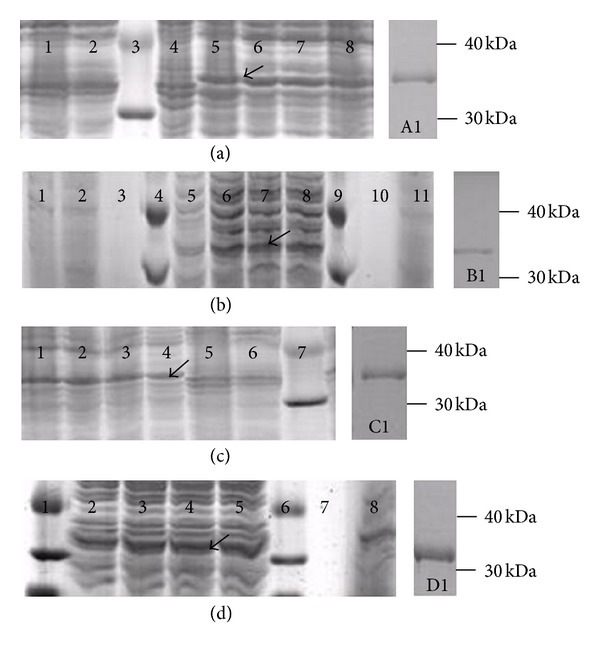
SDS-PAGE analysis of four different recombinant chitinases. (a) Intracellular recombinant chitinase inrCHI31; 1: control transformant BL21-pET52b; 2: control transformant BL21-pET52b induced by 1.0 mM IPTG; 3: protein marker; 4: transformant BL21-inCHI31; 5–8: transformant BL21-inCHI31 induced by 1.0 mM IPTG for 3, 4, 5, and 6 h. (b) Extracellular recombinant chitinase exrCHI31; 1–3: control transformant BL21-pET52b induced by 1.0 mM IPTG for 3, 6, and 0 h; 4, 9: protein marker; 5–8: transformant BL21-exCHI31 induced by IPTG for 3, 4, 5, and 6 h; 10-11: transformant BL21-exCHI31 was not induced. (c) Intracellular recombinant chitinase inrCHI32; 1–5: transformant BL21-inCHI32 induced by 1.0 mM IPTG for 6, 5, 4, 3, and 0 h; 6: control transformant BL21-pET52b induced by 1.0 mM IPTG for 5 h; 7: protein marker. (d) Extracellular recombinant chitinase exrCHI32. 1: protein marker; 2–5: transformant BL21-exCHI32 induced by 1.0 mM IPTG for 3, 4, 5, and 6 h; 6: protein marker; 7, 8: control transformant BL21-pET52b induced for 0 and 3 h at 30°C.

SDS-PAGE analysis was conducted to determine whether the *E. coli* transformants, BL21- inCHI31, BL21-exCHI31, BL21-inCHI32, and BL21-exCHI32, could synthesize recombination chitinase inrCHI31, exrCHI31, inrCHI32, and exrCHI32, respectively. Compared with the control transformant BL21-pET52b, those transformants all showed a clear protein band with a molecular mass of approximately 31 kDa (Figure 2). This result indicated that inrCHI31, exrCHI31, inrCHI32, and exrCHI32 proteins had been successfully synthesized in *E. coli* BL21 strain, and exrCHI31 and exrCHI32 were also secreted into the culture medium. The four recombinant chitinases had also successfully been purified (Figure 2).

### 3.3. Enzymatic Properties

**Figure 3:**
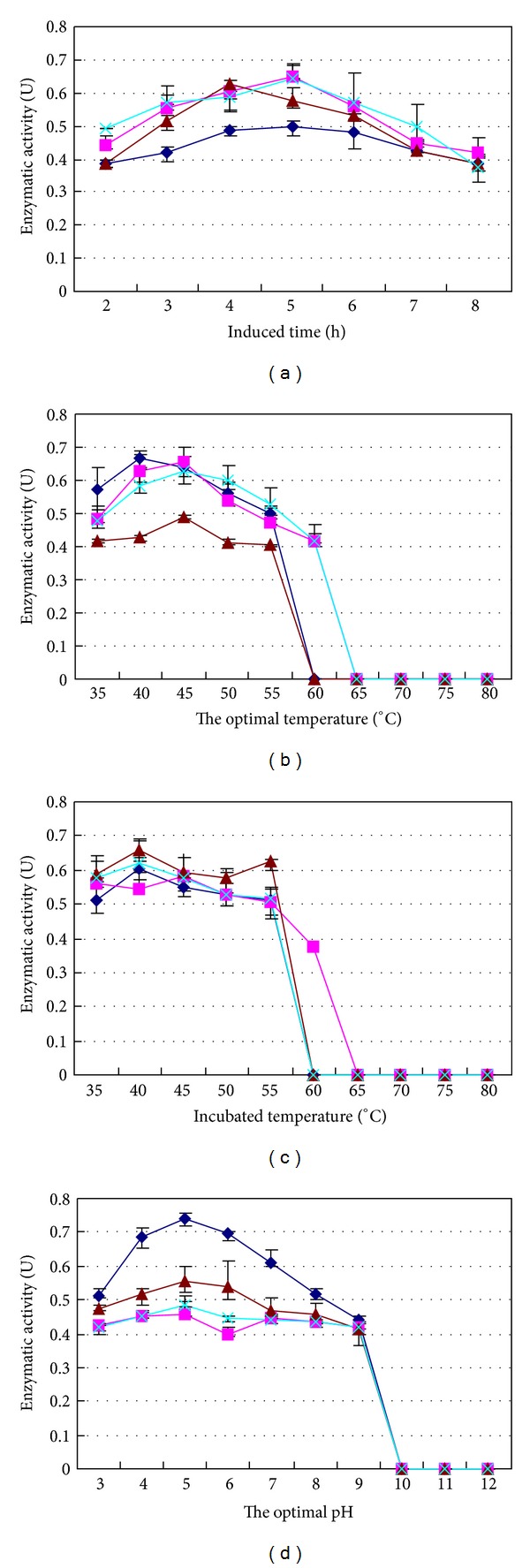
Enzymatic properties of four different recombinant chitinases. Dark blue: enzymatic properties of recombinant intracellular inrCHI31; pink: enzymatic properties of recombinant extracellular exrCHI31; purple: enzymatic properties of recombinant intracellular inrCHI32; Powder blue: enzymatic properties of recombinant intracellular exrCHI32. The precipitates of transformants BL21-inCHI31 and BL21-inCHI32 were used to measure chitinase activity. The culture supernatants of transformants BL21-exCHI31 and BL21-exCHI32 were used to measure chitinase activity. The supernatant was used as a control after being boiled at 100°C for 20 min. (a) Regulation of enzyme production of four transformants BL21-inCHI31, BL21-exCHI31, BL21-inCHI32, and BL21-exCHI32. The four transformants were induced by 1 mM IPTG at 30°C for 2 to 8 h, with 1 h intervals. The enzyme activities at different induced times were measured. (b) The effects of temperature on four recombinant activities. The chitinase activities were measured between 35°C and 80°C for 30 min at 5°C intervals (pH = 4.5). (c) The effects of temperature on the stability of four recombinases. The enzymatic solution (pH = 4.5) was incubated between 35°C and 80°C for 30 min at 5°C intervals, and the remaining activity was then measured. (d) The effects of pH on four recombinase enzymatic activities. Enzymatic activity was measured in the reaction buffer at different pH values from 3 to 12 at 1-unit intervals. All experiments were performed three times.

The activities of chitinases inrCHI31 and exrCHI31 in *E. coli* both showed a peak activity at 5 h following IPTG induction (Figure 3(a)). Moreover, the activities of chitinases inrCHI32 and exrCHI32 in *E. coli* showed a peak activity at 4 and 5 h following IPTG induction, respectively (Figure 3(a)). Chitinase activity was not detected in the culture medium of control *E. coli* BL21-pET52 after IPTG induction, which indicated that the chitinase activity displayed by *E. coli* cells was due to the expression of exogenous *LbCHI31* or *LbCHI32*.

The optimal reaction temperature and pH for intracellular recombinant inrCHI31 was 40°C at a pH of 5.0. Simultaneously, the optimal reaction condition for the activity of prokaryotic extracellular exrCHI31 was 40°C at pH 5.0. Correspondingly, the optimal condition was 45°C at pH 5.0 for recombinant inrCHI32 and 45°C at pH 5.0 for recombinant exrCHI32.

### 3.4. The Effects of Different Ion Levels on Enzymatic Activity

**Figure 4:**
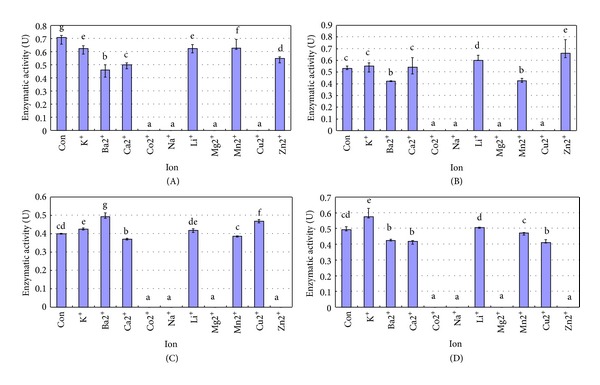
Effects of exposure to different metal ions on the activities of four different recombinases. (A) Activities of recombinase inrCHI31 with different metal ions; (B) activities of recombinase exrCHI31 with different metal ions; (C) activities of recombinase inrCHI32 with different metal ions; (D) activities of recombinase exrCHI32 in the presence of different metal ions. Several different reaction buffers were prepared, each spiked with 5 mmol L^−1^ of each metal ion. The chitinase activities were measured at 37°C for 20 min. Con: control; the activities of inrCHI31, exrCHI31, inrCHI32, and exrCHI32 were measured under normal reaction conditions without any additional ions. The experiments were performed three times. Different letters above the columns indicated a significant difference as determined by Duncan's multiple comparisons test (*P* < 0.05).

The activities of recombinant inrCHI31, exrCHI31, inrCHI32, and exrCHI32 were all strongly inhibited by Co^2+^, Na^+^, and Mg^2+^ (Figure 4). At the same time, inrCHI31 and exrCHI31 were also inhibited by Cu^2+^, and inrCHI32 and exrCHI32 were inhibited by Zn^2+^. In particular, the activity of exrCHI31 was stimulated by Li^+^ and Mn^2+^, respectively (Figure 4(b)). The activity of inrCHI32 was stimulated by K^+^, Ba^2+^, and Cu^2+^ (Figure 4(c)) and the activity of exrCHI32 was stimulated by K^+^ (Figure 4(d)).

### 3.5. The Decomposing Ability to Different Substrates

**Figure 5:**
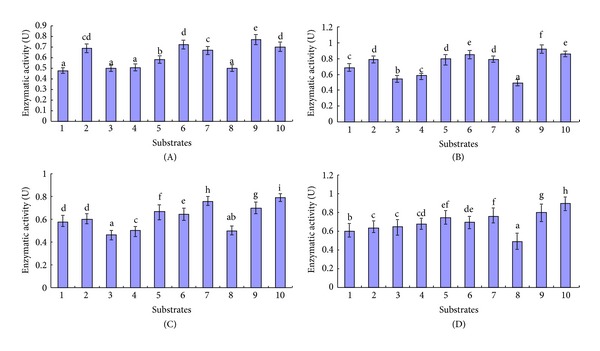
The activities of four different recombinant chitinases on various substrates. (A) Activities of recombinase inrCHI31 to various substrates; (B) activities of recombinase exrCHI31 to various substrates; (C) activities of recombinase inrCHI32 to various substrates; (D) activities of recombinase exrCHI32 to various substrates. One percent (w/v) of substrate was dissolved in 0.02 mol L^−1^ HAc-NaAc buffer (pH 4.5) as reaction solution. Subsamples of the reaction solution were collected and used to measure chitinase activity. Substrates: 1, N.O-carb-chitin; 2, colloidal chitin; 3, chitosan; 4, N.O-carb-chitosan; 5–10, cell wall chitin of *S. sclerotiorum, F. oxysporum, R. solani, P. sojae, A. alternata,* and *V. sordida*. The experiments were performed three times. Different letters above the columns indicated a significant difference determined by Duncan's multiple comparisons test (*P* < 0.05).

The activities of four different recombinant chitinases, inrCHI31, exrCHI31, inrCHI32, and exrCHI32, towards different substrates were measured using Schales method [15]. While four recombinant chitinases demonstrated activity as a response to all test substrates, analysis of variance showed that their decomposing activities towards different substrates were significantly different (*P* < 0.05) (Figure 5). As shown in Figure 5, the decomposed abilities of recombinant chitinases exrCHI31 and exrCHI32 to different substrates were generally higher than those of inrCHI31 and inrCHI32 higher than those of inrCHI31 and inrCHI32. Furthermore, the abilities of four recombinant chitinases to decompose fungal cell walls (Figure 5, lines 6, 7, 9, and 10) were also obviously higher than those of the plant oomycete phytopathogen *P. sojae* (Figure 5, line 8) and chitin derivatives (Figure 5, lines 1–5).

The optimal reaction system of inrCHI31 appears to occur when the temperature was 40°C (Figure 3(b)), the pH was 5.0 (Figure 3(d)), and Mn^2+^ was present at 5 mmol L^−1^ (Figure 4). According to this reaction system, the highest activity achieved by inrCHI31 was 0.772 U when cell wall chitin of the fungal pathogen *A. alternata* was used as a substrate. The optimal reaction system of exrCHI31 appears to occur when the temperature was at 40°C (Figure 4(b)) with a pH value of 5.0 (Figure 3(d)) and 5  mmol L^−1^ of Zn^2+^ (Figure 4). According to this reaction system, the highest activity achieved by exrCHI31 was 0.921 U when cell wall chitin of the fungal pathogen *A. alternata* was used as a substrate. The optimal reaction system of inrCHI32 appeared to occur when the temperature was at 45°C (Figure 3(b)), the pH was 5.0 (Figure 3(d)), and Ba^2+^ was present at 5 mmol L^−1^ (Figure 4). According to the reaction system, the highest activity achieved by inrCHI32 was 0.792 U using cell wall chitin of the fungal pathogen *V. sordida* as a substrate. The optimal reaction system of recombinant exrCHI32 appeared to occur when the temperature was 45°C (Figure 3(b)), the pH was 5.0 (Figure 3(d)), and K^+^ was present at 5 mmol L^−1^ (Figure 4). According to the reaction system, the highest activity achieved by exrCHI32 was 0.897 U when cell wall chitin of the fungal pathogen *V. sordida* was used as the substrate.

### 3.6. Antifungal Activity Analysis of the Recombinant Chitinases

**Figure 6:**
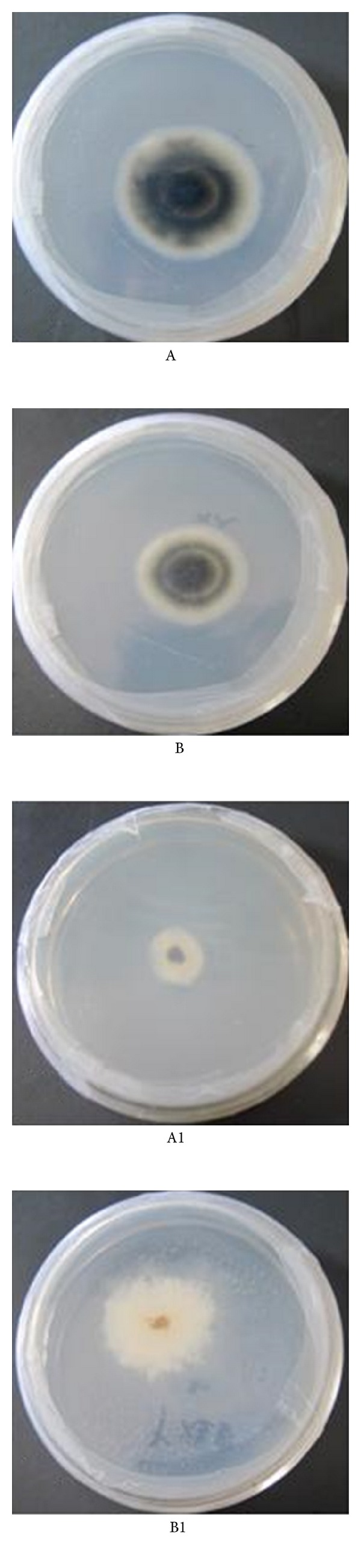
Inhibition of recombinant exrCHI31 (A) and exrCHI32 (B) to *A. alternate*. (A) The mycelia of *A. alternate* grown on PDA medium for 7 d (control); (A1). The mycelia of *A. alternate* grown on PDA medium containing exrCHI31 for 7 d; (B). The mycelia of *A. alternate* grown on PDA medium for 5 d (control); (B1). The mycelia of *A. alternate* grown on PDA medium containing exrCHI32 for 5 d.

Chitinases exrCHI31 and exrCHI32 obviously showed inhibition of the mycelia growth and sporulation of *A. alternata* by plate test. Although recombinant exrCHI31 inhibited the mycelia growth (Figure 6(A)), exrCHI32 inhibited sporulation of *A. alternate* (Figure 6(B)).

## 4. Discussion 

Plant chitinases are pathogenesis-related proteins that are involved in plant defense responses to pathogen infection [16]. The three-dimensional structural model of wheat chitinase showed the presence of 10 *α*-helices, three *β*-strands, 21 loop turns, and six cysteine residues that are responsible for the formation of three disulphide bridges. The active site residues (Glu94 and Glu103) may be responsible for its antifungal activity [10]. The chitinase genes *LbCHI31* (Glu93 and Glu106) and *LbCHI32* (Glu128 and Glu150) (Figure 1) also contain the same structure and active site residues, respectively.

The chitin binding domain (ChtBD1) is a lectin domain found in proteins from plants and fungi that bind *N*-acetylglucosamine and plant endochitinases. This domain is involved in the recognition and/or binding of chitin subunits; it typically occurs towards the N-terminal of glycosyl hydrolase domains in chitinases, together with other carbohydrate-binding domains or by itself in tandem-repeat arrangements. *Brassica juncea* BjCHI1, which is a plant chitinase with two (almost identical) chitin-binding domains, agglutinates Gram-negative bacteria, adversely affecting their growth. In contrast, BjCHI1 derivatives that lack one or both domains do not show agglutination activity, which suggests that both chitin-binding domains are essential for agglutination [17–19]. Usually, the chitinase that contains a chitin-binding domain displays a higher enzyme activity than those without a chitin-binding domain [20]. However, in this study, although recombinant LbCHI31 contains a chitin-binding domain and LbCHI32 does not have this domain, the peak enzymatic activities of these two recombinant chitinases had no visible differences (Figure 5). The reasons that underlie this finding require further investigation.

Chitinase has different antifungal activities on different pathogenic fungi *in vitro*. For example, Pichia-expressed rice chitinase has a different antifungal activity against four fungi: *Rhizopus stolonifer, Botrytis squamosa, Pythium aphanidermatum*, and *Aspergillus niger*. An analysis with scanning electron microscopy (SEM) and Fourier transform infrared spectroscopy (FTIR) showed that this chitinase exhibited different antifungal activities against the four fungi, which was directly correlated to the surface microstructure and the proportion of chitin in the fungal cell wall [11]. In our research, recombinant CHI31 and CHI32 had a high ability to decompose fungal phytopathogen cell walls; however, they had lower decomposing abilities towards oomycota *P. sojae*. The possible reason for this phenomenon is that these six different phytopathogens have different structure cell wall chitins, which means that the same enzyme displayed different decomposing ability to each.

The expression of chitinase (33 kDa) was confirmed by SDS-PAGE and Western hybridization analyses [10]. The yield of purified chitinase was 20 mg/L with a chitinase activity of 1.9 U/mg [10]. As a result of its innate antifungal potential, wheat chitinase can be used to enhance fungal resistance in crop plants [10]. The purified recombinant papaya chitinase CpCHI showed an optimal reaction temperature at 30°C and a broad optimal pH that ranged from 5.0 to 9.0 [11]. In the present study, all the four recombinant chitinases, inrCHI31, exrCHI31, inrCHI32, and exrCHI32, also showed a broad optimal pH that ranged from 3.0 to 9.0, which suggested that chitinases can work well at a wide range of pH.

The enzymatic activity of recombinant intracellular exrCHI31 is higher than that of extracellular inrCHI31 (Figure 5). Similarly, the enzymatic activity of recombinant extracellular exrCHI32 is also higher than that of intracellular inrCHI32 (Figure 5). The probable reason for this phenomenon is that the resolving ability of extracellular recombinant chitinase is higher than that of intracellular recombinant chitinase. Furthermore, extracellular recombinant chitinase could be purified more easily than intracellular recombinant chitinase. Therefore, extracellular recombinant chitinase may have more application values in the future.

This study will aid our understanding of the antifungal mechanism of recombinant chitinases and further determine their scope of applications on crop protection and the postharvest storage of fruits and vegetables.

## Figures and Tables

**Table 1:** The primers used in cloning the *LbCHI31* and *LbCHI32 *genes into prokaryotic intracellular and extracellular expression vectors.

Primers names	Primers sequences (5′-3′)	Underlined enzymatic sites
inCHI31-R	ATCGGGTACCAGGAGCAGTGCGGTTCTCAAGCCGGT	*Kpn*I
inCHI31-L	CGATGAGCTCAGCAAAAGGCCTCTGGCTATTGC	*Sac*I
inCHI32-R	ATCGCCCGGGCTGGACCTGACGGACCAGCTCGTT	*Sma*I
inCHI32-L	CGATGGATCCTGTGACGATGCAGAGCCGGATGGGTT	*Bam*HI
exCHI31-R	ATCGGAAGACTGC**ATG**AAAACGACACTCATCCTAACCG	*Bbs*I
exCHI31-L	CGATGGTACCTCAAGCAAAAGGCCTCTGGCTATTG	*Kpn*I
exCHI32-R	ATCGCC **ATG** GGGAGGCATTGGAGACTGGTAATC	*Nco*I
exCHI32-L	CGATGGTACCTCAATTATGACGATGCAGAGCCGGAT	*Kpn*I
